# Association of Serum Adiponectin Biomarker with Metabolic Syndrome Components in Koreans with Extremely High HDL Cholesterol Levels in General Health Checkup

**DOI:** 10.3390/metabo12111086

**Published:** 2022-11-09

**Authors:** Hyun Suk Yang, Gun-Hyuk Lee, Donghwan Kim, Kyeong Ryong Lee, Mina Hur

**Affiliations:** 1 Department of Cardiovascular Medicine, Konkuk University School of Medicine, Seoul 05030, Korea; 2 Department of Laboratory Medicine, Konkuk University School of Medicine, Seoul 05030, Korea; 3 Research Institute of Medical Science, Konkuk University School of Medicine, Seoul 05030, Korea; 4 Department of Emergency Medicine, Konkuk University School of Medicine, Seoul 05030, Korea

**Keywords:** adiponectin, metabolic syndrome, biomarkers

## Abstract

Adiponectin and high-density lipoprotein cholesterol (HDL-C) are negative predictors for cardio-metabolic disorders. This study explored adiponectin’s role in predicting multiple metabolic syndrome components (multi-MetSC) in subjects with extremely high HDL-C levels overall and by sex. We enrolled adults with extremely high HDL-C levels (≥90 mg/dL) in general health checkups and compared adiponectin levels in subjects with and without multi-MetSC. Among 274 subjects (median 44 years, female 79.6%), 19 (6.9%) had a multi-MetSC. The adiponectin level was significantly lower in subjects with multi-MetSC than without (females: 9.2 [6.2–13.3] vs. 12.0 [9.7–15.9] µg/mL, *p* = 0.039; males: 6.9 ± 2.4 vs. 10.0 ± 5.2 µg/mL, *p* = 0.013). The optimal cutoff values to predict multi-MetSC were 9.7 µg/mL (sensitivity 64%, specificity 74%) in females and 9.6 µg/mL (sensitivity 100%, specificity 44%) in males. Compared with the high adiponectin group, the low group revealed higher fasting glucose in females and higher waist circumference, visceral fat area, and HDL-C levels in males. Multiple logistic regression analysis confirmed adiponectin as an independent predictor of multi-MetSC (OR 0.85, 95% CI 0.71–0.97). Adiponectin could be a potential biomarker for multi-MetSC in general health checkup subjects with extremely high HDL-C levels. There were sex differences in the metabolic risk factors between low and high adiponectin groups.

## 1. Introduction

Metabolic syndrome (MetS) is a cluster of multi-dimensional cardiovascular risk factors that increase the chance of developing type 2 diabetes or cardiovascular diseases. The definition has been formulated from the 1999 World Health Organization, 2001 National Cholesterol Education Program (NCEP) Adult Treatment Panel III (ATP-III), 2002 European Group for the study of insulin resistance, and 2009 International Diabetes Federation formulation [1,2,3,4,5]. The prevalence of MetS ranges from 15% to 35% in adults with various demographics and diagnostic criteria, being more common in men than in women (in Asians, a prevalence of 21% in men, 15% in women; in Koreans, 28% in men, 19% in women) [6,7,8]. The most widely used guideline is the NCEP ATP-III, which includes five components: (1) Abdominal obesity: waist circumference ≥102 cm (men) or ≥88 cm (women), or for Asian, ≥90 cm (men) or ≥80 cm (women); (2) blood pressure (BP) ≥130/85 mmHg or medicated; (3) fasting plasma glucose (FPS) ≥100 mg/dL or medicated; (4) high-density lipoprotein cholesterol (HDL-C) <40 mg/dL (men) or <50 mg/dL (women) or medicated; (5) triglycerides ≥150 mg/dL or medicated [2]. Considering the key features of the MetS are associated with insulin resistance, it may be valuable to explore some simple blood-based biomarkers emphasizing insulin resistance in a general health checkup package.

An adipocyte-derived cytokine, adiponectin could be a blood-based biomarker to reflect MetS components, with primary insulin sensitization, anti-inflammatory, and anti-atherogenic properties [9]. Epidemiological studies showed a positive correlation between adiponectin and HDL-C, suggesting both are negative predictors of MetS as well as atherosclerotic cardiovascular diseases [10,11,12], although their relationship has not been explored for extremely high HDL-C levels where there are paradoxical increases in cardiovascular or all-cause mortalities [13,14,15,16]. The diagnostic accuracy of adiponectin for MetS has been confirmed in a Korean population [17,18], but the adoption of this new supplementary blood test in routine health checkups has lacked sufficient evidence to prove additional benefits on top of the current blood tests, which include glucose, HDL-C, and triglycerides. Moreover, the usefulness of adiponectin as a metabolic risk predictor of low or normal HDL-C levels has been reported [17,18,19], but the usefulness of adiponectin in subjects with extremely high HDL-C levels has not been fully elucidated.

The purpose of this study is to explore the association of the serum adiponectin biomarker with multiple MetS components (multi-MetSC, ≥2 components) in subjects undergoing general health checkups with extremely high HDL-C levels, overall and by sex. In detail, we explored the correlation between serum adiponectin levels and parameters reflecting MetS components and tested whether serum adiponectin is an independent predictor for multi-MetSC among other general health checkup blood variables.

## 2. Materials and Methods

### 2.1. Study Design and Population

This was a retrospective cross-sectional study in a single tertiary medical center. Between April and September 2017, we included consecutive adults (age ≥19 years) with extremely high HDL-C (≥90 mg/dL) in a general health check-up program which includes body composition analysis and adiponectin measurement. We excluded subjects with a medical history of cardiovascular diseases. We retrospectively reviewed their medical records, including routine check-up results and self-administered questionnaires. In this study, multi-MetS was defined as the presence of any two of the following four components based on the NCEP ATP-III with Asian modification [2,7]: (1) Abdominal obesity: waist circumference ≥90 cm (men) or ≥80 cm (women); (2) BP ≥130/85 mmHg or medicated; (3) FPG ≥100 mg/dL or medicated; (4) triglycerides ≥150 mg/dL or medicated.

### 2.2. Anthropometric and Body Composition Measurements

All subjects underwent routine anthropometric measurements, including BP, body weight, height, and waist and hip circumferences. BMI (kg/m^2^) was calculated by dividing body weight (kg) by height squared (m^2^) [20]. Body composition was analyzed using InBody770 (InBody Co., Ltd., Seoul, Korea) based on the direct segmental multi-frequency bioelectrical impedance method [21], determining the parameters of body fat mass (kg), body fat (%), and visceral fat area (cm^2^).

### 2.3. Laboratory Analysis

After at least 12 h of overnight fasting, blood samples were drawn as the routine GME protocol and were centrifuged within one hour. The serum samples were stored at −80 °C until use. Total cholesterol, low-density lipoprotein cholesterol, HDL-C, and triglycerides were measured by enzymatic reagents (Kyowa Kirin Co., Ltd., Tokyo, Japan) on a chemistry analyzer (TBA-200FR NEO, Toshiba Medical System Co., Tokyo, Japan). Low-density lipoprotein cholesterol (LDL-C) was calculated using the Friedewald formula: LDL-C = Total cholesterol-(triglycerides/5)-HDL-C. Serum adiponectin was measured by Randox immunoturbidimetric adiponectin assay (Randox Laboratories Ltd., Crumlin, UK), according to the manufacturer’s protocol [22].

### 2.4. Statistical Analysis

Data are expressed as frequency (percentage) for categorical variables or a mean ± standard deviation or median with the interquartile range for continuous variables. All continuous data were tested for normal distribution using Kolmogorov-Smirnov nonparametric tests. We performed comparison analysis in two groups with Chi-square or Fisher’s exact test (categorical variables) and an independent T-test or Mann–Whitney U test (continuous variables) in four groups with the Kruskal–Wallis test with Turkey’s post hoc test. We assessed the correlation between adiponectin levels and continuous variables using Pearson’s correlation coefficient (r). The correlation was interpreted as negligible (<0.1), weak (0.1–0.39), moderate (0.40–0.69), strong (0.70–0.89), or very strong (≥0.9) based on the absolute magnitude [23]. We analyzed the ROC curves for serum adiponectin levels to predict multi-MetSC. Finally, we performed the univariate binary and multiple logistic regression analysis of blood variables reflecting metabolic syndrome components or lipid profiles for predicting multi-MetSC (*p*-value threshold of 0.2 for multiple regression, likelihood method for CI). All the statistical analysis was performed by web-based analysis with R 4.2 (http://web-r.org, accessed on 5 June 2022). The level of significance (alpha) was set to 0.05 (two-tailed).

## 3. Results

### 3.1. Characteristics of the Study Population

Characteristics of the 274 subjects overall and by sex are presented in Table 1. The median age was 44 [37–51] years with females comprising 79.6%. The median body mass index (BMI) was 20.8 [19.3–22.6] kg/m^2^; triglycerides, 57.5 [45.0–75.0] mg/dL; and adiponectin, 11.6 [8.5–14.7] µg/mL. Females had significantly lower BP, FPS, liver enzymes, BMI, waist circumference, and visceral fat areas but a higher total and total percentage of body fat. The serum adiponectin level was significantly higher in females than in males (12.0 [9.4–15.6] vs. 8.6 [6.2–12.0] µg/mL, *p* < 0.001). Females revealed lower rates of MetS components (high BP, high FPS, and multi-MetSC) than males (*p* < 0.05).

Among the 218 females, 53 (24.3%) were postmenopausal. The serum adiponectin level was significantly higher in post- than in pre-menopausal females (14.0 [10.6–18.0] vs. 11.7 [9.1–14.6] µg/mL, *p* = 0.005) (Supplementary Materials Figure S1).

Hormone model assessment for insulin resistance (HOMA-IR) was available in 97 subjects (females, 80; males, 17), with a median value of 0.94 [0.68–1.29] and no significant difference between females and males (0.95 [0.66–1.25] vs. 0.85 [0.69–1.87], *p* = 0.404).

### 3.2. Correlations between Serum Adiponectin Levels and Parameters Reflecting MetS Components

The Pearson’s correlation coefficients between adiponectin levels and parameters reflecting MetS components or lipid profiles are shown in Table 2. In all subjects, serum adiponectin levels showed a weak negative correlation with BMI, waist circumference, waist-to-hip ratio, body fat mass, visceral fat area, FPG, and triglycerides.

### 3.3. Comparison of Serum Adiponectin Levels with or without Multi-MetSC

**Figure 1:**
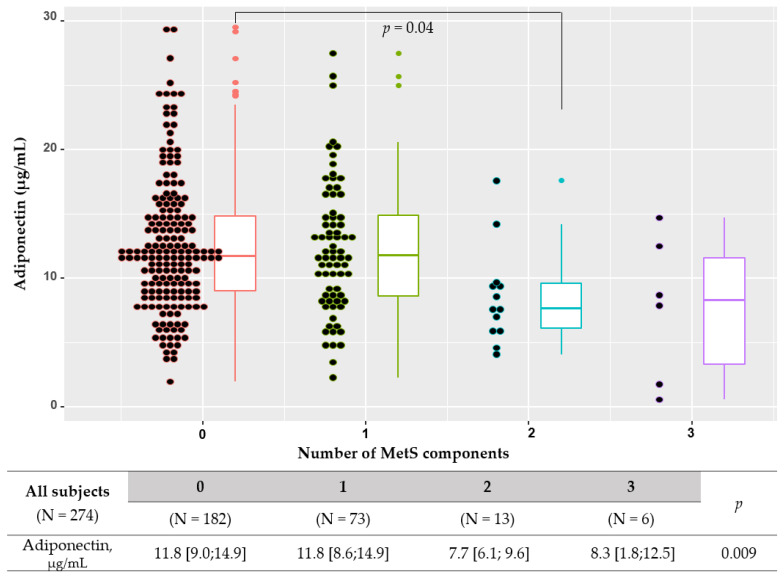
Box-and-whisker dot plots. Four group comparisons of serum adiponectin levels according to the number of metabolic syndrome (MetS) components. Kruskal–Wallis test with Turkey’s post hoc test was used: (0 vs. 1) *p* = 0.99, (0 vs. 2) *p* = 0.04, (0 vs. 3) *p* = 0.11, (1 vs. 2) *p* = 0.075, (1 vs. 3) *p* = 0.15, and (2 vs. 3) *p* = 0.99.

**Figure 2:**
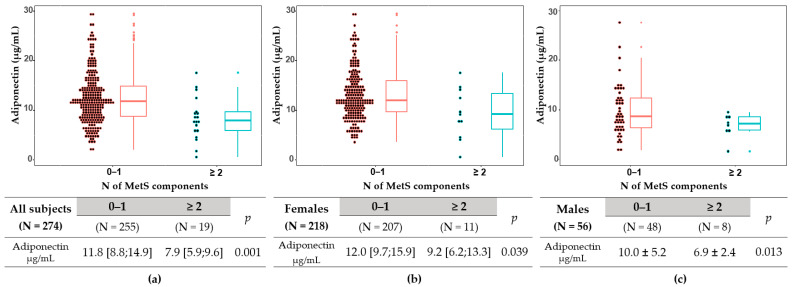
Box-and-whisker dot plots. Two group comparisons of serum adiponectin levels with or without multiple metabolic syndrome components (≥2) in all subjects (**a**), females (**b**), and males (**c**). An independent two-sample test (*t*-test or Mann–Whitney test) was used after the normality test.

Of the 274 subjects, no MetS component was seen in 182 (66.4%), one component in 73 (26.6%), two components in 13 (4.7%), three components in 6 (2.2%), and four components in 0 (0.0%) (Table 1, Figure 1). Multi-MetSC (≥2 MetS components) was observed in 19 (6.9%) subjects, more frequent in males than in females (14.3% vs. 5.0%, *p* = 0.015). Adiponectin levels were significantly lower in subjects with multi-MetS than in those without (all subjects: 7.9 [5.9–9.6] vs. 11.8 [8.8–14.9] μg/mL, *p* = 0.001; females: 9.2 [6.2–13.3] vs. 12.0 [9.7–15.9] μg/mL, *p* = 0.039; males: 6.9 ± 2.4 vs. 10.0 ± 5.2 μg/mL, *p* = 0.013) (Figure 2).

### 3.4. Optimal Cutoff Values of Adiponectin to Predict Multi-MetSC

**Figure 3:**
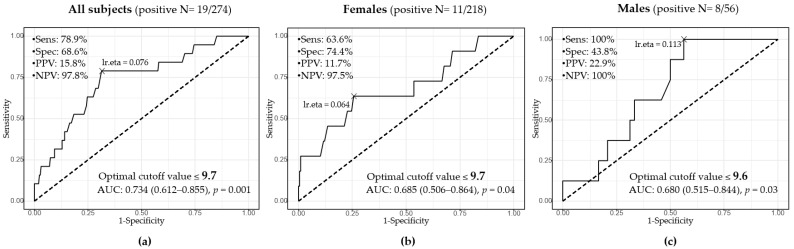
The receiver operating characteristic curves for serum adiponectin levels to predict multi-metabolic syndrome components ((**a**): All subjects, (**b**): Females, and (**c**): Males). The area under the curve (AUC) with a 95% confidence interval is shown in each group. The optimal cutoff value by the Youden index is presented with sensitivity (Sens), specificity (Spec), positive predictive value (PPV), and negative predictive value (NPV).

The receiver-operating characteristic (ROC) curves demonstrated that serum adiponectin levels could predict multi-MetSC in all subjects and by sex, and the optimal cutoff values are shown in Figure 3. In all subjects, adiponectin showed an area under the curve (AUC) of 0.743 (*p* = 0.001) with a cutoff of ≤9.7 µg/mL, sensitivity of 79%, and specificity of 69%. In females, there was an AUC of 0.685 (*p* = 0.04) with a cutoff of ≤9.7 µg/mL; in males, an AUC of 0.680 (*p* = 0.03) with a cutoff of ≤9.6 µg/mL.

### 3.5. Comparison of General Health Checkup Parameters between Low and High Adiponectin Groups

The characteristics of subjects with low and high adiponectin levels (according to the cutoff values in Figure 3) are presented in Table 3. The low group showed significantly higher body weight, BMI, waist circumference, waist-to-hip ratio, and visceral fat area than the high group both in all subjects and in males (*p* < 0.05), though not in females (*p* > 0.3). The low group revealed significantly higher FPG than the high group in all subjects and females (*p* < 0.05).

### 3.6. Logistic Regression Analysis of Blood Variables including Adiponectin for Predicting Multi-MetSC

In univariate binary logistic regression analysis, adiponectin (odds ratio [OR] 0.81 with 95% confidence interval [CI] 0.70–0.91, *p* < 0.001), FPS (OR 1.16, 95% CI 1.10–1.24, *p* < 0.001), and HbA1c (OR 1.16, 95% CI 2.02–18.4, *p* = 0.002) were associated with multi-MetSC. Multiple logistic regression analysis confirmed adiponectin as a significant negative predictor for multi-MetSC (OR 0.85. 95% CI 0.69–0.96, *p* = 0.031) for all subjects (Table 4).

**Table 3:** Comparison of general health checkup parameters between the low and high serum adiponectin level groups.

	All Subjects			Females			Males		
	Low Group N = 95	High Group N = 179	*p*	Low Group N = 60	High Group N = 158	*p*	Low Group N = 35	High Group N = 21	*p* Value
Adiponectin, μg/mL	7.7 [5.8;8.6]	13.7 [11.7;17.1]	**<0.001**	7.9 [5.8;8.8]	13.8 [11.7;17.2]	**<0.001**	6.9 [5.5;8.1]	13.5 [11.8;14.9]	**<0.001**
Age, years	45.0 [38.0;49.5]	44.0 [37.0;51.0]	0.75	43.0 [37.5;47.0]	43.0 [36.0;50.0]	0.67	47.5 ± 9.7	53.0 ± 14.2	0.09
Anthropometic and body composition measurements									
Body weight, kg	57.1 [50.5;65.0]	53.0 [49.8;57.8]	**0.003**	52.9 [48.5;58.3]	52.8 [49.4;57.2]	0.98	68.3 ± 10.7	61.0 ± 6.4	**0.002**
BMI, kg/m^2^	21.2 [19.8;23.2]	20.6 [19.1;22.1]	**0.014**	20.6 [19.4;22.2]	20.6 [19.1;22.1]	0.68	22.9 [21.1;24.4]	20.6 [19.3;22.3]	**0.002**
Waist/hip ratio	0.83 ± 0.06	0.80 ± 0.06	**<0.001**	0.81 [0.77;0.83]	0.81 [0.75;0.84]	0.49	0.89 ± 0.05	0.84 ± 0.06	**0.008**
Body fat mass, kg	13.9 [12.0;16.5]	13.7 [11.8;16.0]	0.50	13.9 [12.4;16.5]	14.2 [12.2;16.9]	1.00	13.9 [10.8;16.5]	9.5 [8.1;12.5]	**0.001**
Body fat, %	24.7 ± 5.8	25.7 ± 6.3	0.17	27.2 ± 4.6	27.1 ± 5.2	0.80	20.2 ± 5.0	15.7 ± 3.8	**0.001**
VFA, cm^2^	59.0 [43.0;91.5]	50.0 [33.0;68.0]	**0.005**	48.0 [38.0;65.0]	48.0 [32.0;66.0]	0.70	89.0 ± 29.7	71.1 ± 26.7	**0.028**
Laboratory analysis									
Hemoglobin, mg/dL	14.1 [13.1;14.9]	13.4 [12.8;14.0]	**<0.001**	13.6 [12.6;14.1]	13.2 [12.6;13.8]	0.08	15.2 ± 1.1	14.9 ± 0.9	0.32
HbA1c, %	5.3 [5.1;5.5]	5.3 [5.2;5.5]	0.79	5.3 [5.1;5.5]	5.3 [5.2;5.5]	0.97	5.3 [5.2;5.5]	5.4 [5.3;5.6]	0.30
Total-C, mg/dL	212.0 [191.5;233.0]	214.0 [196.5;235.5]	0.37	212.3 ± 26.6	218.1 ± 27.6	0.17	217.8 ± 32.2	212.5 ± 30.9	0.55
LDL-C, mg/dL	104.0 [83.5;124.0]	105.0 [86.5;121.0]	0.47	103.1 ± 25.7	107.4 ± 25.9	0.28	105.0 [82.0;128.0]	101.0 [89.0;119.0]	0.87
AST, mg/dL	22.0 [19.0;28.5]	22.0 [19.0;26.0]	0.31	20.0 [18.0;23.0]	21.0 [18.0;25.0]	0.18	28.0 [23.5;37.5]	26.0 [23.0;31.0]	0.38
ALT, mg/dL	15.0 [12.0;24.5]	15.0 [12.0;20.0]	0.13	14.0 [11.0;18.0]	15.0 [11.0;18.0]	0.64	22.0 [15.5;32.5]	22.0 [19.0;25.0]	0.75
γGT, mg/dL	22.0 [14.0;41.5]	16.0 [13.0;24.0]	**<0.001**	17.0 [12.5;23.5]	16.0 [12.0;21.0]	0.51	46.0 [26.0;63.5]	27.0 [21.0;37.0]	**0.028**
TB, mg/dL	0.9 [0.7;1.2]	0.8 [0.6;1.0]	**0.009**	0.8 [0.6;1.0]	0.8 [0.6;1.0]	0.97	1.1 [1.0;1.4]	1.1 [0.8;1.3]	0.44
Creatinine, mg/dL	0.8 [0.7;0.9]	0.8 [0.7;0.8]	**0.025**	0.7 [0.7;0.8]	0.8 [0.7;0.8]	0.14	1.0 ± 0.1	1.0 ± 0.1	0.56
GFR, mL/min/1.73 m^2^	86.0 [78.0;91.0]	84.0 [76.0;91.0]	0.14	87.5 [79.5;91.0]	84.0 [76.0;91.0]	**0.039**	81.0 [76.5;89.5]	79.0 [76.0;90.0]	0.77
Metabolic syndrome components reflecting variables									
WC, cm	74.5 [68.5;80.2]	71.0 [67.0;75.0]	**<0.001**	71.0 [67.5;76.0]	70.5 [66.5;75.0]	0.31	80.0 [75.0;86.8]	74.5 [70.5;78.5]	**0.006**
Systolic BP, mmHg	120.0 [108.0;128.0]	113.0 [102.0;120.0]	**0.007**	112.5 [103.0;123.0]	110.0 [102.0;120.0]	0.48	124.9 ± 10.4	122.6 ± 9.9	0.42
Diastolic BP, mmHg	72.0 [65.0;79.0]	69.0 [61.0;79.0]	0.08	70.0 [63.0;76.0]	69.0 [61.0;78.0]	0.71	76.7 ± 9.7	74.7 ± 10.2	0.46
FPG, mg/dL	93.0 [86.5;96.5]	87.0 [82.0;91.5]	**<0.001**	91.5 [85.5;94.5]	87.0 [82.0;91.0]	**0.001**	94.0 [87.5;102.5]	90.0 [82.0;96.0]	0.10
HDL-C, mg/dL	95.0 [93.0;99.0]	94.0 [92.0;98.0]	0.13	95.0 [93.0;97.0]	94.0 [92.0;98.0]	0.42	96.7 ± 4.9	94.0 ± 3.0	**0.014**
Triglycerides, mg/dL	61.0 [48.0;79.0]	56.0 [43.0;73.0]	0.054	59.5 [48.0;82.0]	56.0 [43.0;73.0]	0.07	64.0 [49.0;76.5]	55.0 [49.0;70.0]	0.52
Metabolic syndrome components									
Abdominal obesity	12 (12.6%)	18 (10.1%)	0.66	7 (11.7%)	18 (11.4%)	1.00	5 (14.3%)	0 (0.0%)	0.15
High BP	26 (27.4%)	30 (16.8%)	0.055	9 (15.0%)	22 (13.9%)	1.00	17 (48.6%)	8 (38.1%)	0.63
High FPG	18 (18.9%)	12 (6.7%)	**0.004**	8 (13.3%)	8 (5.1%)	**0.045**	10 (28.6%)	4 (19.0%)	0.63
High triglycerides	1 (1.1%)	0 (0.0%)	0.35	1 (1.7%)	0 (0.0%)	0.28	0 (0.0%)	0 (0.0%)	0.35
≥2 components	15 (15.8%)	4 (2.2%)	**<0.001**	7 (11.7%)	4 (2.5%)	**0.011**	8 (22.9%)	0 (0.0%)	**0.020**
≥3 components	4 (4.2%)	2 (1.1%)	0.19	2 (3.3%)	2 (1.3%)	0.30	2 (5.7%)	0 (0.0%)	0.52

Values are expressed as median [interquartile range], mean ± standard deviation, or number (percentage). Mann–Whitney U or independent *t*-test (quantitative variables) and Chi-square or Fisher’s exact test (categorical variables) were used; bold figures indicate *p* < 0.05. The low (≤9.7 µg/mL) and high (>9.7 µg/mL) adiponectin level groups in total subjects and females; the low (≤9.6 µg/mL) and high (>9.6 µg/mL) in males. Abbreviations: see Table 1.

## 4. Discussion

In this extremely high HDL-C level cohort, subjects with multi-MetSC showed significantly lower serum adiponectin levels than those without, in all subjects and by sex. The association of adiponectin levels and MetS components has been reported as a global systematic review or meta-analysis [26,27] and as a Korean study by sex [28]. For the Korean reference values, two large-scale studies were reported. Park et al. reported adiponectin references as 13.4 [9.3–18.9] µg/mL in females and 8.2 [6.2–11.0] µg/mL in males via the enzyme-linked immunosorbent assay (ELISA) method with a Korean health checkup cohort [29]. Koh et al. reported adiponectin references as 12.82 [9.58–16.70] µg/mL in females and 8.90 [6.24–12.74] µg/mL in males via radioimmunoassay [28]. As a cross-sectional study, low adiponectin’s association with MetS has been reported in apparently healthy subjects (MetS vs. no MetS: females 5.7 ± 1.8 vs. 8.6 ± 2.1 µg/mL, *p* < 0.001; males 3.3 ± 1.8 vs. 4.4 ± 2.1 µg/mL, *p* = 0.002) by ELISA [18], in non-diabetic adults (MetS vs. no MetS: females 10.12 [7.62–13.41] vs. 11.74 [8.80–15.22] µg/mL, *p* < 0.001; males 6.00 [4.32–8.53] vs. 8.00 [5.62–11.33] µg/mL, *p* < 0.001) by radioimmunoassay [28], and in farmers (MetS vs. no MetS: females 7.8 ± 3.2 vs. 9.6 ± 3.8 µg/mL; males 5.1 ± 2.1 vs. 7.6 ± 3.2 µg/mL, all *p* < 0.05) by ELISA [30]. As a prospective study, the baseline adiponectin levels predicted de-novo MetS after a median 2.4-year follow-up (MetS vs. no MetS: females 11.0 [7.8–14.8] vs. 12.2 [9.1–15.5] µg/mL, *p* < 0.001; males 7.1 [4.9–9.5] vs. 8.6 [6.3–11.7] µg/mL, *p* < 0.001) by radioimmunoassay [17].

Our study is unique in terms of the study subjects, including Koreans with extremely high HDL-C levels (≥90 mg/dL) in general health checkups. Koh et al. studied non-diabetic Koreans, reporting baseline HDL-C levels as 47.4 ± 11.0 mg/dL in females and 46.2 ± 11.7 mg/dL in males, which are low to normal [28]. Compared to several Korean studies with low to normal HDL-C levels in healthy volunteers, our baseline adiponectin levels measured by immunoturbidimetric assay are higher than some studies using ELISA [18,30,31] but similar to the two large-scale studies (one by ELISA, the other, radioimmunoassay) [28,29]. Although almost all of the previous studies reported a positive relationship between adiponectin and HDL-C levels [12,19,29,30,32], our study showed no significant correlation between the two in all subjects. Furthermore, in males, the low adiponectin group with a higher rate of multi-MetSC revealed paradoxically higher HDL-C levels than the high group (96.7 ± 4.9 vs. 94.0 ± 3.0 mg/dL, *p* = 0.014), suggesting that low adiponectin levels could predict metabolic risk even in subjects with extremely high HDL-C levels. For females, we should consider using a level of 130 mg/dL as the “extremely high” threshold since the cardiovascular risk level at 130 is similar to that of 90 in males in a Korean population [16]. Unfortunately, with our study subjects, the median value of adiponectin levels was 94.7 [92.0–98.0] µg/mL with a range of 90–133 µg/mL in females, which may not fully represent the true extremely high HDL-C levels (≥130 mg/dL). For the mechanism related to adiponectin and HDL-C, a specific single nucleotide polymorphism has been reported [33] but the metabolic association between the two is considered to be regulated by a complex genetic pathway and environmental factors [34]. The positive correlation between adiponectin and HDL-C levels has been explained by adiponectin-induced up-regulation of HDL-C: adiponectin increases HDL-C via hepatic production of apo-AI and ATP-binding cassette transporter A1 [35] or via activation of lipoprotein lipase [36]. However, in this study, the mechanism regarding the paradoxical relationship between adiponectin and HDL-C levels in males with extremely high HDL-C levels is not clear. We only speculate that heavy alcohol intake may have played a role. Subjects with extremely high HDL-C levels tend to show heavy alcohol drinking, which may be related to lower serum adiponectin levels in Korean men [16,37]. Therefore, considering the mutual effects between adiponectin and HDL-C during the metabolic risk evaluation is reasonable, especially in subjects with extremely high HDL-C levels.

In this study, the optimal cutoff value to predict multi-MetSC was 9.7 µg/mL (sensitivity 79%, specificity 69%) for all subjects, 9.7 µg/mL (sensitivity 64%, specificity 74%) in females, and 9.6 µg/mL (sensitivity 100%, specificity 44%) in males. The relatively high sensitivity seems good for health screening purposes. Similar to the previous studies, our study demonstrated the independent association of low adiponectin levels with multi-MetSC in all subjects (OR 0.85, 95% CI 0.71–0.97, *p* = 0.031). By sex, there was a trend but not enough statistical significance (females: OR 0.85, 95% CI 0.70–1.00, *p* = 0.065; males: OR 0.71, 95% CI 0.39–1.01, *p* = 0.14) in multiple logistic regression with blood variables reflecting MetS components or lipid profiles, which may be due to the low number of study subjects. Pathophysiological roles of adiponectin for multi-MetSC have been suggested. Mendelian randomization studies were inconsistent for the causality, one supporting an adiponectin causal relationship to insulin sensitivity [38], another not supporting causation of insulin resistance or type 2 diabetes [39]. The Adipo Q gene, from which human adiponectin is encoded, is located on chromosomes 3 and 17, which is well-established for influence on phenotypes of MetS [40]. The adiponectin level’s inverse relationship with the presence of MetS is considered to be mediated by insulin sensitivity, anti-inflammatory, and anti-atherosclerotic effects [9,38].

Here, we presented sex-specific characteristics of the low vs. high adiponectin groups in subjects with extremely high HDL-C levels, which were not presented in previous studies. In males, the low adiponectin group had a significantly higher waist circumference and visceral fat area, as we expected. In females, however, the expected obese phenotype was not distinguishable, but elevated FPG was significant in the low adiponectin group compared with the high adiponectin group. Sex differences in the progression of metabolic risk factors have been emphasized in diabetes development [41], and sex-specific biomarker levels should be considered in future studies [16].

This study has several limitations. First, it was a small cross-sectional study. Second, some inquiries about family history, lifestyle, and nutritional habits are missing data. Third, female subgroup analysis by the status of menopause was limited (Supplementary Materials), with a lack of information about long-term hormone replacement therapy or oral contraceptives containing different progestogens. There have been complex reports regarding the influence of adiponectin levels on menopausal status, hormone replacement therapy, or contraception [42,43,44]. Fourth, the target outcome was multi-MetSC (≥two components out of four) instead of the more robust MetS (≥three components out of five) or major adverse cardiovascular events. Even though the MetS components may be easy to assess in a clinical setting, it would be important to learn if the adiponectin level can help to determine the atherosclerotic risk for subjects with extremely high HDL-C. Therefore, in the future, a larger prospective study with solid outcomes (atherosclerotic cardiovascular diseases or all-cause or cardiovascular mortality) analyzing HDL-subclasses [45] might be useful to assess adiponectin’s role as a biomarker for metabolic or atherosclerotic cardiovascular diseases.

## 5. Conclusions

Adiponectin showed the potential to be an incremental blood-based biomarker for multi-MetSC in subjects with extremely high HDL-C levels in general medical checkups. Sex differences were noted in the metabolic risk factors between the low and high adiponectin groups.

## Figures and Tables

**Table 1:** Characteristics of the study population.

	All Subjects N = 274	Females N = 218	Males N = 56	*p*
Age, years	44.0 [37.0;51.0]	43.0 [36.0;48.0]	49.0 [41.5;55.0]	**<0.001**
Family history *				
Hypertension	111 (46.6%)	89 (46.6%)	22 (46.8%)	0.98
Diabetes mellitus	60 (25.2%)	50 (26.2%)	10 (21.3%)	0.49
Smoking ^†^				
Never	196 (80.0%)	183 (95.8%)	13 (24.1%)	**<0.001**
Past smoker	29 (11.8%)	6 (3.1%)	23 (42.6%)	
Current smoker	20 (8.2%)	2 (1.0%)	18 (33.3%)	
Alcohol consumption ^‡^				
None	84 (31.3%)	81 (38.2%)	3 (5.4%)	**<0.001**
Adequate	137 (51.1%)	113 (53.3%)	24 (42.9%)	
Intermediate	39 (14.6%)	17 (8.0%)	22 (39.3%)	
Heavy alcohol use	8 (3.0%)	1 (0.5%)	7 (12.5%)	
Exercise frequency ^§^				
0/week	98 (35.8%)	83 (38.1%)	15 (26.8%)	0.095
1–4/week	111 (40.5%)	89 (40.8%)	22 (39.3%)	
≥5/week	65 (23.7%)	46 (21.1%)	19 (33.9%)	
Anthropometric and body composition measurements				
Weight, kg	53.8 [49.9;61.0]	52.8 [49.1;57.3]	65.0 [57.8;71.4]	**<0.001**
BMI, kg/m^2^	20.8 [19.3;22.6]	20.6 [19.2;22.1]	22.1 [20.1;23.4]	**0.005**
WC, cm	72.0 [67.5;78.0]	70.8 [67.0;75.0]	78.2 [74.0;83.5]	**<0.001**
Waist/hip ratio	0.82 [0.77;0.85]	0.81 [0.76;0.84]	0.89 [0.83;0.91]	**<0.001**
Body fat mass, kg	13.9 [11.9;16.1]	14.0 [12.2;16.9]	12.4 [8.5;15.1]	**<0.001**
Body fat, %	25.4 ± 6.1	27.1 ± 5.0	18.5 ± 5.1	**<0.001**
VFA, cm^2^	52.0 [36.0;74.0]	48.0 [34.0;66.0]	85.0 [62.5;101.0]	**<0.001**
Systolic BP, mmHg	116.0 [104.0;124.0]	111.5 [102.0;120.0]	125.0 [117.5;132.5]	**<0.001**
Diastoic BP, mmHg	71.0 [63.0;79.0]	69.0 [61.0;77.0]	76.0 [68.5;80.5]	**<0.001**
Laboratory analysis				
Hemoglobin, mg/dL	13.5 [12.9;14.3]	13.3 [12.6;13.8]	15.1 [14.6;15.6]	**<0.001**
FPG, mg/dL	89.0 [83.0;94.0]	87.5 [82.0;93.0]	93.0 [87.0;99.0]	**<0.001**
HbA1c, %	5.3 [5.1;5.5]	5.3 [5.1;5.5]	5.4 [5.2;5.5]	0.36
Total-C, mg/dL	214.0 [195.0;234.0]	214.0 [196.0;234.0]	215.5 [188.5;233.0]	0.76
LDL-C, mg/dL	105.0 [85.0;123.0]	105.0 [85.0;121.0]	104.5 [83.0;126.0]	0.92
HDL-C, mg/dL	95.0 [92.0;98.0]	95.0 [92.0;98.0]	95.0 [91.5;99.0]	0.86
Triglycerides, mg/dL	57.5 [45.0;75.0]	57.5 [44.0;75.0]	57.5 [48.0;73.5]	0.63
AST, mg/dL	22.0 [19.0;26.0]	21.0 [18.0;25.0]	27.5 [23.0;37.0]	**<0.001**
ALT, mg/dL	15.0 [12.0;21.0]	15.0 [11.0;18.0]	22.0 [16.5;32.0]	**<0.001**
γGT, mg/dL	18.0 [13.0;27.0]	16.0 [12.0;22.0]	36.5 [23.5;60.0]	**<0.001**
TB, mg/dL	0.8 [0.7;1.1]	0.8 [0.6;1.0]	1.1 [1.0;1.4]	**<0.001**
Creatinine, mg/dL	0.8 [0.7;0.9]	0.8 [0.7;0.8]	1.0 [0.9;1.1]	**<0.001**
GFR, mL/min/1.73 m^2^	84.0 [76.0;91.0]	85.0 [77.0;91.0]	80.5 [76.0;90.0]	0.25
Adiponectin, μg/mL	11.6 [8.5;14.7]	12.0 [9.4;15.6]	8.6 [6.2;12.0]	**<0.001**
Medications				
Hypertension	10 (3.6%)	4 (1.8%)	6 (10.7%)	**0.006**
Diabetes mellitus	4 (1.5%)	0 (0.0%)	4 (7.1%)	**0.002**
Dyslipidemia	13 (4.7%)	8 (3.7%)	5 (8.9%)	0.15
Number of Metabolic syndrome components ¶				
**0**	182 (66.4%)	160 (73.4%)	22 (39.3%)	**<0.001**
**1**	73 (26.6%)	47 (21.6%)	26 (46.4%)	**<0.001**
Abdominal obesity	30 (10.9%)	25 (11.5%)	5 (8.9%)	0.76
High BP	56 (20.4%)	31 (14.2%)	25 (44.6%)	**<0.001**
High FPG	30 (10.9%)	16 (7.3%)	14 (25.0%)	**<0.001**
High triglycerides	1 (0.4%)	1 (0.5%)	0 (0.0%)	1.00
**2**	13 (4.7%)	7 (3.2%)	6 (10.7%)	**0.018**
Abd + BP	4 (1.5%)	2 (0.9%)	2 (3.6%)	0.19
Abd + FPG	2 (0.7%)	2 (0.9%)	0 (0.0%)	1.00
BP + FPG	6 (2.1%)	2 (0.9%)	4 (7.1%)	**0.017**
FPG + TG	1 (0.4%)	1 (0.5%)	0 (0.0%)	1.00
**3** Abd + BP + FPG	6 (2.2%)	4 (1.8%)	2 (3.6%)	0.61

Values are expressed as median [interquartile range], mean ± standard deviation, or number (percentage). *p* is based on the comparison between females and males using the Mann–Whitney U or independent *t*-test (quantitative variables) and Chi-square or Fisher’s exact test (categorical variables); bold figures indicate *p* < 0.05. * Data were available for 238 subjects (females, 191; males, 47). ^†^ Data were available for 245 subjects (females, 191; males, 54). ^‡^ Data were available for 268 subjects (females, 212; males, 56). Adequate is defined as one standard drink (14 g of ethanol, as found in five ounces of wine or 12 ounces of beer) per day or seven standard drinks per week with no more than three drinks per occasion. Heavy alcohol use is defined as binge drinking (four drinks for women and five drinks for men) on five or more days in the past month [24]. ^§^ One time means ≥30 min of moderate-intensity aerobic physical activity (3–5.9 MET, for example walking at moderate or brisk pace, slow cycling, gardening, golf, tennis, or ballroom dancing [25]. ¶ NCEP ATP-III with Asian modification: (1) Abdominal obesity: waist circumference ≥90 cm (men) or ≥80 cm (women); (2) BP ≥ 130/85 mmHg or medicated; (3) FPG ≥ 100 mg/dL or medicated; (4) HDL-C < 40 mg/dL (men) or <50 mg/dL (women) or medicated; (5) triglycerides ≥ 150 mg/dL or medicated. Abbreviations: BP, blood pressure; BMI, body mass index; WC, waist circumference; VFA, visceral fat area; FPG, fasting plasma glucose; HbA1c, glycated hemoglobin; Total-C, total cholesterol; LDL-C, low-density lipoprotein cholesterol; HDL-C, high-density lipoprotein cholesterol; AST, aspartate transaminase; ALT, alanine transaminase; γGT, gamma-glutamyl transferase; TB, total bilirubin; GFR, glomerular filtration rate based on the Modification of Diet in Renal Disease Study equation; Abd, abdominal obesity.

**Table 2:** Correlations between adiponectin levels and parameters reflecting MetS components or lipid profiles.

	All Subjects N = 274		Females N = 218		Males N = 56	
	r	*p*	r	*p*	r	*p*
BMI, kg/m^2^	−0.255	**<0.0001**	−0.170	**0.012**	−0.389	**0.003**
WC, cm	−0.307	**<0.0001**	−0.200	**0.003**	−0.339	**0.011**
Waist/hip ratio	−0.247	**<0.0001**	−0.119	0.080	−0.252	0.061
Body fat mass, kg	−0.129	**0.032**	−0.140	**0.039**	−0.373	**0.005**
Body fat, %	0.029	0.629	−0.094	0.167	−0.358	**0.007**
VFA, cm^2^	−0.217	**<0.0001**	−0.100	0.140	−0.227	0.093
Systolic BP, mmHg	−0.102	0.093	−0.008	0.907	−0.096	0.612
Diastolic BP, mmHg	−0.113	0.063	−0.034	0.620	−0.136	0.319
FPG, mg/dL	−0.191	**0.002**	−0.160	**0.018**	−0.079	0.565
HbA1c, %	−0.006	0.916	0.017	0.806	0.063	0.647
Total-C, mg/dL	0.062	0.310	0.073	0.286	0.021	0.877
LDL-C, mg/dL	0.027	0.651	0.031	0.647	0.049	0.722
HDL-C, mg/dL	0.041	0.496	0.061	0.370	−0.100	0.464
Triglycerides, mg/dL	−0.131	**0.030**	−0.128	0.058	−0.140	0.304

Pearson’s correlation coefficient (r). Bold figures indicate *p* < 0.05. Variables: see Table 1.

**Table 4:** Logistic regression analysis of blood variables reflecting metabolic syndrome components or lipid profiles for predicting multi-MetSC in subjects with extremely high HDL-C levels.

	All Subjects				Females				Males			
	Univariate		Multivariable		Univariate		Multivariable		Univariate		Multivariable	
	OR (95% CI)	*p*	OR (95% CI)	*p*	OR (95% CI)	*p*	OR (95% CI)	*p*	OR (95% CI)	*p*	OR (95% CI)	*p*
Adiponectin	0.81 (0.70–0.91)	**<0.001**	0.85 (0.71–0.97)	**0.031**	0.82 (0.69–0.96)	**0.019**	0.85 (0.70–1.00)	0.065	0.83 (0.64–1.01)	0.103	0.71 (0.39–1.01)	0.144
FPG	1.16 (1.10–1.24)	**<0.001**	1.20 (1.11–1.31)	**<0.001**	1.24 (1.13–1.39)	**<0.001**	1.24 (1.12–1.41)	**<0.001**	1.10 (1.04–1.20)	**0.006**	1.29 (1.07–1.74)	**0.031**
HbA1c	5.62 (2.02–18.4)	**0.002**	0.26 (0.04–1.66)	0.161	6.50 (0.85–50.6)	0.069	1.14 (0.10–13.4)	0.917	3.87 (1.24–16.9)	**0.037**	0.03 (0.00–1.58)	0.131
Total-C	1.02 (1.00–1.03)	0.065	1.03 (0.96–1.10)	0.329	1.01 (0.99–1.04)	0.244			1.02 (0.99–1.04)	0.152	1.03 (0.99–1.07)	0.126
LDL-C	1.01 (1.00–1.03)	0.098	0.98 (0.92–1.06)	0.597	1.01 (0.99–1.04)	0.293			1.02 (0.99–1.05)	0.226		
HDL-C	0.98 (0.88–1.06)	0.626			0.97 (0.84–1.07)	0.659			0.99 (0.82–1.16)	0.882		
Triglycerides	1.01 (1.00–1.03)	0.090	1.01 (0.99–1.03)	0.380	1.01 (0.99–1.03)	0.284			1.02 (0.99–1.05)	0.190	1.01 (0.97–1.05)	0.636

Bold figures indicate *p*< 0.05. OR, odds ratio; CI, confidence interval. Variables: see Table 1.

## Data Availability

All the data described in this study are available within the article or its supplementary materials.

## Supplementary Materials

The following supporting information can be downloaded at https://www.mdpi.com/article/10.3390/metabo12111086/s1, Figure S1: Comparison of serum adiponectin levels between pre- and post-menopausal females; Figure S2: Comparisons of serum adiponectin levels with or without multiple metabolic syndrome components in pre-menopausal females and post-menopausal females; Figure S3: The receiver operating characteristic curves for serum adiponectin levels to predict multi-metabolic syndrome components; Table S1: Logistic regression analysis of blood variables reflecting metabolic syndrome components or lipid profiles for predicting multi-MetSC in pre-menopausal females with extremely high HDL-C levels.

## References

[1] Alberti K.G., Zimmet P.Z. (1998). Definition, diagnosis and classification of diabetes mellitus and its complications. Part 1: Diagnosis and classification of diabetes mellitus provisional report of a WHO consultation. Diabet. Med..

[2] (2001). Executive summary of the third report of the national cholesterol education program (NCEP) expert panel on detection, evaluation, and treatment of high blood cholesterol in adults (Adult Treatment Panel III). JAMA.

[3] Balkau B., Charles M.A., Drivsholm T., Borch-Johnsen K., Wareham N., Yudkin J.S., Morris R., Zavaroni I., van Dam R., Feskins E. (2002). Frequency of the WHO metabolic syndrome in European cohorts, and an alternative definition of an insulin resistance syndrome. Diabetes Metab..

[4] Alberti K.G., Eckel R.H., Grundy S.M., Zimmet P.Z., Cleeman J.I., Donato K.A., Fruchart J.C., James W.P., Loria C.M., Smith S.C. (2009). Harmonizing the metabolic syndrome: A joint interim statement of the International Diabetes Federation Task Force on Epidemiology and Prevention; National Heart, Lung, and Blood Institute; American Heart Association; World Heart Federation; International Atherosclerosis Society; and International Association for the Study of Obesity. Circulation.

[5] Alshammary A.F., Alharbi K.K., Alshehri N.J., Vennu V., Ali Khan I. (2021). Metabolic syndrome and coronary artery disease risk: A meta-analysis of observational studies. Int. J. Environ. Res. Public Health.

[6] Hirode G., Wong R.J. (2020). Trends in the prevalence of metabolic syndrome in the United States, 2011–2016. JAMA.

[7] Tan C.E., Ma S., Wai D., Chew S.K., Tai E.S. (2004). Can we apply the National Cholesterol Education Program Adult Treatment Panel definition of the metabolic syndrome to Asians?. Diabetes Care.

[8] Kim M.-h., Lee S.-H., Shin K.-S., Son D.-Y., Kim S.-H., Joe H., Yoo B.-W., Hong S.-H., Cho C.-Y., Shin H.-S. (2020). The change of metabolic syndrome prevalence and its risk factors in Korean adults for decade: Korea national health and nutrition examination survey for 2008–2017. KJFP.

[9] Jung H.N., Jung C.H. (2021). The Role of anti-inflammatory adipokines in cardiometabolic disorders: Moving beyond adiponectin. Int. J. Mol. Sci..

[10] Roumeliotis S., Liakopoulos V., Roumeliotis A., Stamou A., Panagoutsos S., D’Arrigo G., Tripepi G. (2021). Mutual effect modification between adiponectin and HDL as risk factors of cardiovascular events in type 2 diabetes individuals: A cohort study. Int. Urol. Nephrol..

[11] Jang A.Y., Scherer P.E., Kim J.Y., Lim S., Koh K.K. (2021). Adiponectin and cardiometabolic trait and mortality: Where do we go?. Cardiovasc. Res..

[12] Yamamoto Y., Hirose H., Saito I., Tomita M., Taniyama M., Matsubara K., Okazaki Y., Ishii T., Nishikai K., Saruta T. (2002). Correlation of the adipocyte-derived protein adiponectin with insulin resistance index and serum high-density lipoprotein-cholesterol, independent of body mass index, in the Japanese population. Clin. Sci..

[13] Ko D.T., Alter D.A., Guo H., Koh M., Lau G., Austin P.C., Booth G.L., Hogg W., Jackevicius C.A., Lee D.S. (2016). High-density lipoprotein cholesterol and cause-specific mortality in individuals without previous cardiovascular conditions: The CANHEART study. J. Am. Coll. Cardiol..

[14] Madsen C.M., Varbo A., Nordestgaard B.G. (2017). Extreme high high-density lipoprotein cholesterol is paradoxically associated with high mortality in men and women: Two prospective cohort studies. Eur. Heart J..

[15] Hirata A., Sugiyama D., Watanabe M., Tamakoshi A., Iso H., Kotani K., Kiyama M., Yamada M., Ishikawa S., Murakami Y. (2018). Association of extremely high levels of high-density lipoprotein cholesterol with cardiovascular mortality in a pooled analysis of 9 cohort studies including 43,407 individuals: The EPOCH-JAPAN study. J. Clin. Lipidol..

[16] Yang H.S., Jeong H.J., Kim H., Hwang H.K., Hur M., Lee S. (2022). Sex-specific U-shaped relationships between high-density lipoprotein cholesterol levels and 10-year major adverse cardiovascular events: A nationwide cohort study of 5.7 million South Koreans. Ann. Lab. Med..

[17] Kim J.Y., Ahn S.V., Yoon J.H., Koh S.B., Yoon J., Yoo B.S., Lee S.H., Park J.K., Choe K.H., Guallar E. (2013). Prospective study of serum adiponectin and incident metabolic syndrome: The ARIRANG study. Diabetes Care.

[18] Yoo K.H., Oi I.M., Park J.E., Kim M.J., Park J.S., Park S.J., Jang E.J., Park S.W., Kim S.J., Yoon Y.S. (2012). Metabolic syndrome is associated with low adiponectin level and increased insulin resistance in apparently healthy Koreans. Korean J. Obes..

[19] Park S.H., Kim J.Y., Lee J.H., Park H.Y. (2010). Association between plasma adiponectin and high-density lipoprotein cholesterol in postmenopausal women. Clin. Biochem..

[20] Alshammary A.F., Khan I.A. (2021). Screening of obese offspring of first-cousin consanguineous subjects for the angiotensin-converting enzyme gene with a 287-bp alu sequence. JOMES.

[21] Lahav Y., Goldstein N., Gepner Y. (2021). Comparison of body composition assessment across body mass index categories by two multifrequency bioelectrical impedance analysis devices and dual-energy X-ray absorptiometry in clinical settings. Eur. J. Clin. Nutr..

[22] (2016). Adiponectin Package Insert, Cat. No. AO 2799, Rev. 005, 55 Diamond Road, Crumlin, Country Antrium, UK.

[23] Schober P., Boer C., Schwarte L.A. (2018). Correlation coefficients: Appropriate use and interpretation. Anesth. Analg..

[24] Patrick M.E., Azar B. (2018). High-Intensity Drinking. Alcohol. Res..

[25] Visseren F.L.J., Mach F., Smulders Y.M., Carballo D., Koskinas K.C., Bäck M., Benetos A., Biffi A., Boavida J.-M., Capodanno D. (2021). 2021 ESC guidelines on cardiovascular disease prevention in clinical practice: Developed by the task force for cardiovascular disease prevention in clinical practice with representatives of the European Society of Cardiology and 12 medical societies with the special contribution of the European Association of Preventive Cardiology (EAPC). Eur. Heart J..

[26] Srikanthan K., Feyh A., Visweshwar H., Shapiro J.I., Sodhi K. (2016). Systematic review of metabolic syndrome biomarkers: A panel for early detection, management, and risk stratification in the West Virginian population. Int. J. Med. Sci..

[27] Li Y., Shi B., Li S. (2014). Association between serum chemerin concentrations and clinical indices in obesity or metabolic syndrome: A meta-analysis. PLoS ONE.

[28] Koh S.B., Yoon J., Kim J.Y., Yoo B.S., Lee S.H., Park J.K., Choe K.H. (2011). Relationships between serum adiponectin with metabolic syndrome and components of metabolic syndrome in non-diabetic Koreans: ARIRANG study. Yonsei Med. J..

[29] Park Y., Kim Y.R., Kim H.S. (2013). Evaluation of the performance of an adiponectin ELISA-based test and establishing serum adiponectin reference intervals for Korean population. Lab. Med. Online.

[30] Yeon S.E., Son H.R., Choi J.S., Kim E.K. (2014). Relationships among serum adiponectin, leptin and vitamin D concentrations and the metabolic syndrome in farmers. Korean J. Community Nutr..

[31] Yoon S.J., Lee H.S., Lee S.W., Yun J.E., Kim S.Y., Cho E.R., Lee S.J., Jee E.J., Lee H.Y., Park J. (2008). The association between adiponectin and diabetes in the Korean population. Metabolism.

[32] Tomono Y., Hiraishi C., Yoshida H. (2018). Age and sex differences in serum adiponectin and its association with lipoprotein fractions. Ann. Clin. Biochem..

[33] Arregui M., Fisher E., Knüppel S., Buijsse B., di Giuseppe R., Fritsche A., Corella D., Willich S.N., Boeing H., Weikert C. (2012). Significant associations of the rs2943634 (2q36.3) genetic polymorphism with adiponectin, high density lipoprotein cholesterol and ischemic stroke. Gene.

[34] Kangas-Kontio T., Huotari A., Ruotsalainen H., Herzig K.H., Tamminen M., Ala-Korpela M., Savolainen M.J., Kakko S. (2010). Genetic and environmental determinants of total and high-molecular weight adiponectin in families with low HDL-cholesterol and early onset coronary heart disease. Atherosclerosis.

[35] Van Linthout S., Foryst-Ludwig A., Spillmann F., Peng J., Feng Y., Meloni M., Van Craeyveld E., Kintscher U., Schultheiss H.P., De Geest B. (2010). Impact of HDL on adipose tissue metabolism and adiponectin expression. Atherosclerosis.

[36] Schneider J.G., von Eynatten M., Schiekofer S., Nawroth P.P., Dugi K.A. (2005). Low plasma adiponectin levels are associated with increased hepatic lipase activity in vivo. Diabetes Care.

[37] Jung S.K., Kim M.K., Shin J., Choi B.Y. (2013). A cross-sectional analysis of the relationship between daily alcohol consumption and serum adiponectin levels among adults aged 40 years or more in a rural area of Korea. Eur. J. Clin. Nutr..

[38] Gao H., Fall T., van Dam R.M., Flyvbjerg A., Zethelius B., Ingelsson E., Hägg S. (2013). Evidence of a causal relationship between adiponectin levels and insulin sensitivity: A Mendelian randomization study. Diabetes.

[39] Yaghootkar H., Lamina C., Scott R.A., Dastani Z., Hivert M.F., Warren L.L., Stancáková A., Buxbaum S.G., Lyytikäinen L.P., Henneman P. (2013). Mendelian randomization studies do not support a causal role for reduced circulating adiponectin levels in insulin resistance and type 2 diabetes. Diabetes.

[40] Kissebah A.H., Sonnenberg G.E., Myklebust J., Goldstein M., Broman K., James R.G., Marks J.A., Krakower G.R., Jacob H.J., Weber J. (2000). Quantitative trait loci on chromosomes 3 and 17 influence phenotypes of the metabolic syndrome. Proc. Natl. Acad. Sci. USA.

[41] Yoshida Y., Chen Z., Baudier R.L., Krousel-Wood M., Anderson A.H., Fonseca V.A., Mauvais-Jarvis F. (2022). Sex differences in the progression of metabolic risk factors in diabetes development. JAMA Netw. Open.

[42] Sieminska L., Wojciechowska C., Niedziolka D., Marek B., Kos-Kudla B., Kajdaniuk D., Nowak M. (2005). Effect of postmenopause and hormone replacement therapy on serum adiponectin levels. Metabolism.

[43] Im J.A., Lee J.W., Lee H.R., Lee D.C. (2006). Plasma adiponectin levels in postmenopausal women with or without long-term hormone therapy. Maturitas.

[44] Di Carlo C., Tommaselli G.A., De Rosa N., Fabozzi A., Santoro R., Bifulco G., Sparice S., Nappi C. (2011). Plasma leptin and adiponectin levels in hormone replacement therapy and contraception: Effects of different progestogens. Fertil. Steril..

[45] Yang H.S., Hur M., Kim H., Kim S.J., Shin S., Di Somma S., Network G. (2020). HDL subclass analysis in predicting metabolic syndrome in Koreans with high HDL cholesterol levels. Ann. Lab. Med..

