# Supplementary material for: Association of Serum Adiponectin Biomarker with Metabolic Syndrome Components in Koreans with Extremely High HDL Cholesterol Levels in General Health Checkup
Source: Metabolites. 2022 Nov 9;12(11):1086. doi: 10.3390/metabo12111086 (PMC9694422; doi:10.3390/metabo12111086)
Supplement: Supplementary file 1 [file metabolites-12-01086-s001.zip › metabolites-1909326-supplementary.pdf]

[Supplementary Materials]

Female subgroup analysis (Pre- and Post-menopausal females)

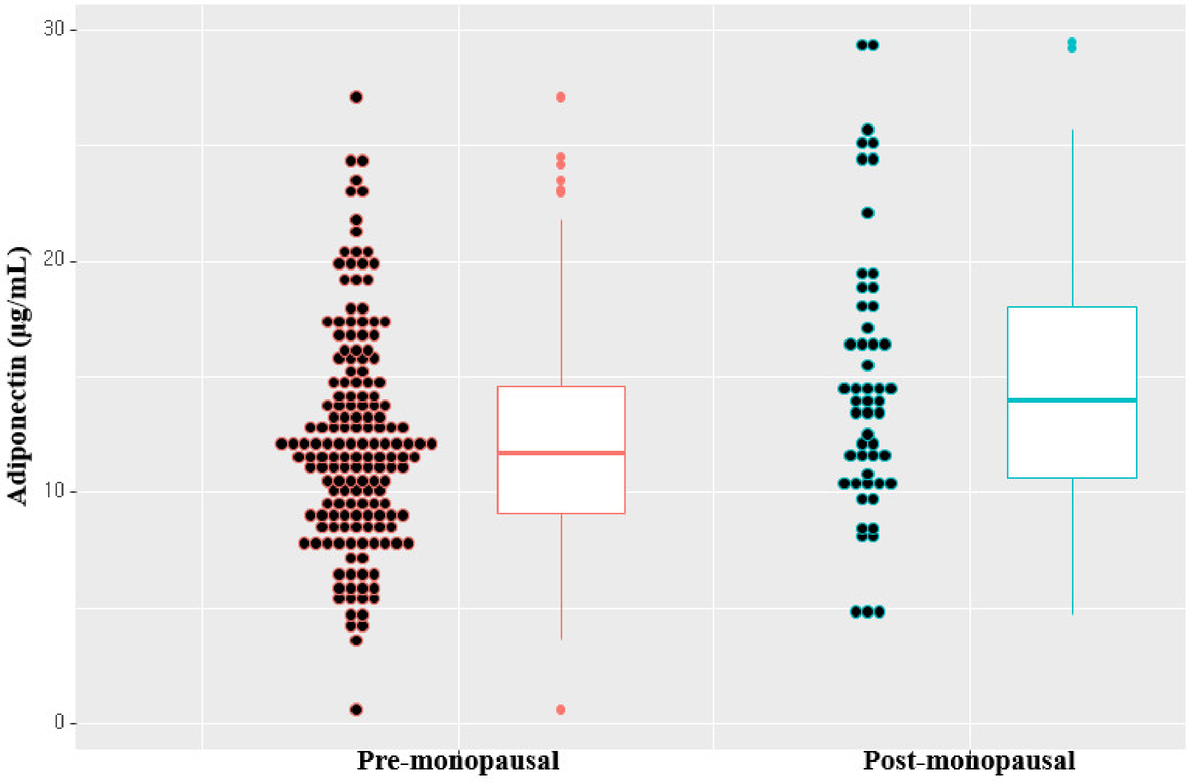

Females (N=218)	Pre-menopausal (N=165)	Post-menopausal (N=53)	*P*
Adiponectin, μg/mL	11.7 [9.1;14.6]	14.0 [10.6;18.0]	0.005

Figure S1. Box-and-whisker dot plots. Comparison of serum adiponectin levels between pre- and post-menopausal females. Mann-Whitney test was applied after the normality test.

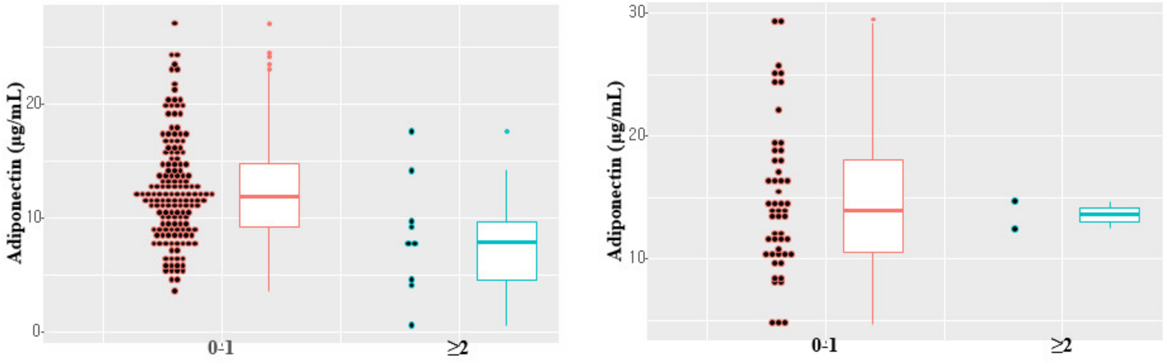

Pre-monopausal (N=165)	0-1 (N=156)	≥ 2 (N=9)	*P*
Adiponectin, μg/mL	11.9 [9.2;14.8]	7.9 [4.6; 9.7]	0.019

Post-monopausal (N=53)	0-1 (N=51)	≥ 2 (N=2)	*P*
Adiponectin, μg/mL	14.0 [10.6;18.1]	13.6 [12.5;14.7]	1.000

(a) (b)

Figure S2. Box-and-whisker dot plots. Comparisons of serum adiponectin levels with or without multiple metabolic syndrome components in pre-menopausal females (a) and post-menopausal females (b). Mann-Whitney test was applied after the normality test.

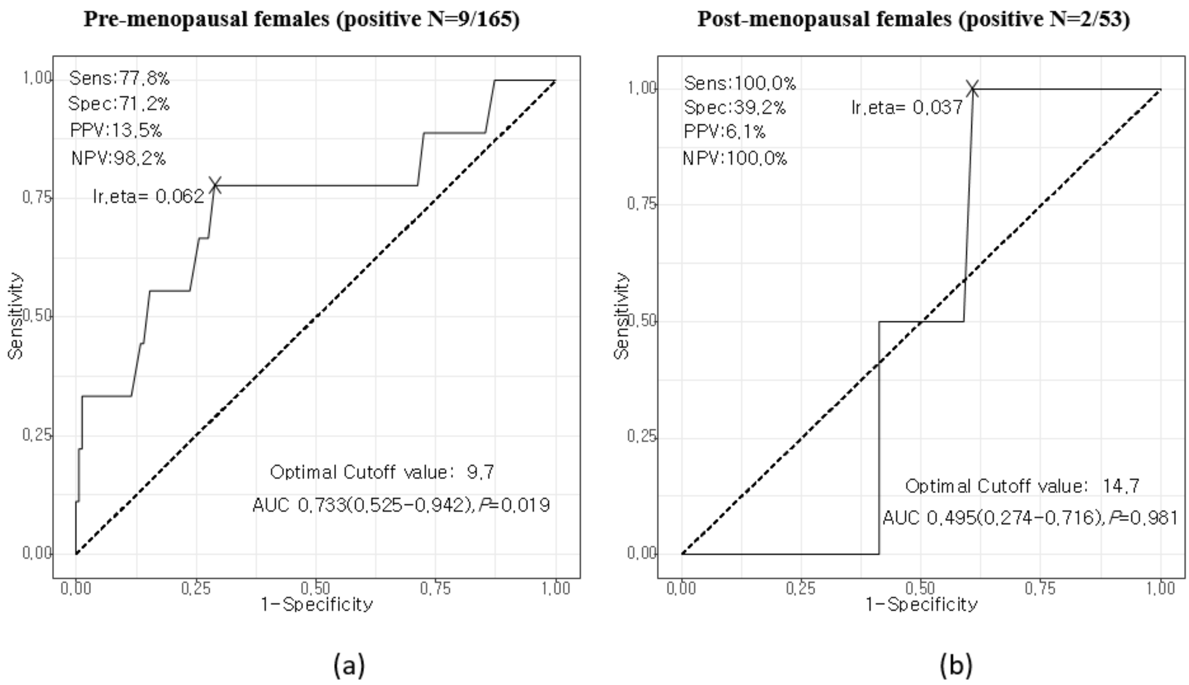

Figure S3. The receiver operating characteristic curves for serum adiponectin levels to predict multi-metabolic syndrome components (a: pre-menopausal females, b: post-menopausal females). The area under the curve (AUC) with a 95% confidence interval is shown in both groups. The optimal cutoff value by the Youden index is presented with sensitivity (Sens), specificity (Spec), positive predictive value (PPV), and negative predictive value (NPV).

Table S1. Logistic regression analysis of blood variables reflecting metabolic syndrome components or lipid profiles for predicting multi-MetSC in pre-menopausal females (n=165) with extremely high HDL-C levels. Bold figures indicate *P*< 0.05. OR, odds ratio; CI, confidence interval. Variables: see Table 1.

	**Univariate**		**Multivariable**	
	OR (95% CI)	*P*	OR (95% CI)	*P*
Adiponectin	0.78 (0.63-0.93)	**0.012**	0.84 (0.68-1.00)	**0.071**
FPG	1.24 (1.12-1.41)	**<0.001**	1.23 (1.10-1.41)	**<0.001**
HbA1c	1.97 (0.17-22.8)	0.583		
Total-C	1.02 (0.99-1.04)	0.224		
LDL-C	1.02 (0.99-1.05)	0.227		
HDL-C	0.99 (0.86-1.08)	0.849		
Triglycerides	1.01 (0.99-1.04)	0.246		
